# Myeloid cell ACE shapes cellular metabolism and function in PCSK-9 induced atherosclerosis

**DOI:** 10.3389/fimmu.2023.1278383

**Published:** 2023-10-20

**Authors:** DuoYao Cao, Suguru Saito, LiMin Xu, Wei Fan, Xiaomo Li, Faizan Ahmed, Predrag Jovanovic, Tomohiro Shibata, Mingtian Che, Ellen A. Bernstein, Jorge Gianni, Ajit S. Divakaruni, Derick Okwan-Duodu, Zakir Khan, Celine E. Riera, Fanfan Chen, Kenneth E. Bernstein

**Affiliations:** ^1^ Department of Biomedical Sciences, Cedars-Sinai Medical Center, Los Angeles, CA, United States; ^2^ Department of Neurosurgery, Shenzhen Entry-Exit Frontier Inspection Hospital, Shenzhen, China; ^3^ Karsh Division of Gastroenterology and Hepatology, Cedars-Sinai Medical Center, Los Angeles, CA, United States; ^4^ Department of Pathology, Cedars-Sinai Medical Center, Los Angeles, CA, United States; ^5^ Biobank and Pathology Shared Resource, Cedars-Sinai Medical Center, Los Angeles, CA, United States; ^6^ Department of Molecular and Medical Pharmacology, University of California, Los Angeles (UCLA) David Geffen School of Medicine, Los Angeles, CA, United States; ^7^ Department of Pathology, Faculty of Medicine, Stanford University, San Jose, CA, United States; ^8^ Department of Neurosurgery, Shenzhen Key Laboratory of Neurosurgery, The First Affiliated Hospital of Shenzhen University, Shenzhen Second People’s Hospital, Shenzhen, China

**Keywords:** atherosclerosis, angiotensin converting enzyme (ACE), monocytes, Ly-6Clo, macrophages, lipid metabolism

## Abstract

The pathogenesis of atherosclerosis is defined by impaired lipid handling by macrophages which increases intracellular lipid accumulation. This dysregulation of macrophages triggers the accumulation of apoptotic cells and chronic inflammation which contributes to disease progression. We previously reported that mice with increased macrophage-specific angiotensin-converting enzyme, termed ACE10/10 mice, resist atherosclerosis in an adeno-associated virus-proprotein convertase subtilisin/kexin type 9 (AAV-PCSK9)-induced model. This is due to increased lipid metabolism by macrophages which contributes to plaque resolution. However, the importance of ACE in peripheral blood monocytes, which are the primary precursors of lesional-infiltrating macrophages, is still unknown in atherosclerosis. Here, we show that the ACE-mediated metabolic phenotype is already triggered in peripheral blood circulating monocytes and that this functional modification is directly transferred to differentiated macrophages in ACE10/10 mice. We found that Ly-6Clo monocytes were increased in atherosclerotic ACE10/10 mice. The monocytes isolated from atherosclerotic ACE10/10 mice showed enhanced lipid metabolism, elevated mitochondrial activity, and increased adenosine triphosphate (ATP) levels which implies that ACE overexpression is already altered in atherosclerosis. Furthermore, we observed increased oxygen consumption (VO2), respiratory exchange ratio (RER), and spontaneous physical activity in ACE10/10 mice compared to WT mice in atherosclerotic conditions, indicating enhanced systemic energy consumption. Thus, ACE overexpression in myeloid lineage cells modifies the metabolic function of peripheral blood circulating monocytes which differentiate to macrophages and protect against atherosclerotic lesion progression due to better lipid metabolism.

## Introduction

Atherosclerosis is a prevalent condition in coronary artery disease and is a leading cause of death worldwide (1). In the immune-pathogenic aspect, the accumulation of macrophages with a high lipid burden due to impaired lipid metabolism, known as foam cells, in the arterial wall is a critical factor in the development and rupture of atherosclerotic plaques (2). Foam cells within atherosclerosis have been observed to experience a loss of function. This includes a reduction in adenosine triphosphate (ATP), consequently decreasing cellular activity, which leads to the failure of eliminating apoptotic cells represented by efferocytosis for neutrophils in the atherosclerotic plaque (3). Therefore, it is essential to understand an efficient approach that promotes lipid metabolism in macrophages and their precursors, monocytes.

Angiotensin-converting enzyme (ACE) is generally known as an enzyme responsible for elevated blood pressure by producing the potent vasoconstrictor Ang II (4). Based on this physiological role, ACE inhibitors often regulate ACE activity in conditions such as hypertension and cardiovascular diseases, including atherosclerosis (4). Moreover, the increased ACE expression in macrophages infiltrating atherosclerotic plaques can serve as a marker to evaluate the severity of atherosclerosis (5, 6). However, our recent findings suggest that enhanced ACE expression might support maintaining the metabolic function of macrophages, thus preventing the progression of atherosclerosis. In fact, we discovered ACE overexpression in macrophages enhances β-oxidation via the activation of the peroxisome proliferator-activated receptor alpha (PPARα)-carnitine palmitoyl transferase (CPT)1A/2 dependent pathway. This enhancement ultimately reduces the formation of atherosclerotic plaques in adeno-associated virus-proprotein convertase subtilisin/kexin type 9 (AAV-PCSK9)-induced atherosclerosis in ACE10/10 mice (7, 8). We further showed that ACE 10/10 macrophages stimulate abundant ATP production dependent on Kreb’s cycle intermediates compared with WT cells (9–12). Hence, our findings suggest that the upregulation of ACE may positively enhance metabolic reactions in macrophages, which ultimately provides a protective effect in atherosclerosis.

While the precise role of enhanced ACE expression in monocytes which are major sources of plaque-infiltrating macrophages in atherosclerosis remains unclear, following our previous findings, we hypothesized that the metabolic characteristics of monocytes are also altered by ACE overexpression, which may be directly reflected in macrophage characteristics after differentiation. In this report, we demonstrate that Ly-6C^lo^ monocytes, which are elevated in the peripheral blood of atherosclerotic ACE10/10 mice, exhibit modified metabolic and anti-inflammatory functions upon differentiation into macrophages. This discovery reveals that the regulation of ACE expression significantly influences the modification of metabolic function across the myeloid lineage.

## Materials and methods

### Animal experiments

The atherosclerosis model was established by following the protocol described in previous reports (7, 11). Briefly, 5x10^10^ copies of AAV-PCSK9 or Null-AAV (No. AAV-268246, 7026, Vector Biolabs) were infected into 6-week-old sex-matched (20 females and 20 males) WT and ACE10/10 mice by intravenous (i.v.) injection (7, 13–15). The mice were then fed with an atherogenic rodent diet (No. D12336i, Research Diets) for 18 weeks unless otherwise indicated. All animal experiments were approved by the Institutional Animal Care and Usage Committee (IACUC) of Cedars-Sinai Medical Center and were performed by following the guidelines of the NIH Guide for the Care and Use of Laboratory Animals.

### Lesional lipid staining

The entire length of the aorta was isolated and fixed in 10% buffered formalin for 12 hours. After fixation, the specimens were rinsed in 70% ethanol for 5 minutes and then stained with Sudan IV for an additional 5 minutes. Following staining, the samples were washed in 80% ethanol. The entire length of the artery was photographed using a digital camera for further analysis. For histological examination, 8 μm-thick frozen sections of the aortic root were prepared and stained with oil red O. The QuPath software (version 0.2.3) was utilized to analyze the lesional area stained for lipids.

### Flow cytometry

Flow cytometry analysis was performed using SONY ID7000 (SONY, Tokyo, Japan) with fluorochrome-conjugated monoclonal antibody (mAb) samples. Briefly, the cells were first treated with FcγRII/III blocker (anti-CD16/CD32) at 4°C for 10 min followed by staining with fluorochrome-conjugated or unconjugated mAb at 4°C for 60 min. The samples for staining with secondary antibody were further incubated with corresponding fluorochrome-conjugated mAb at 4°C for 60 min. To measure metabolic response and mitochondrial activity, the cells were treated with chemical probes at 4°C for 60 min. To analyze apoptotic status, the cells were stained with annexin V. The antibodies and probes used for flow cytometry analysis are listed in **Supplementary Table 1**. All the data were analyzed using FlowJo version 10.4 (BD bioscience, Franklin Lakes, NJ, USA).

### Western blot

Macrophage-derived monocytes (MDMs) were lysed using RIPA buffer containing protease and phosphatase inhibitors (Thermo Fisher Scientific, Waltham, MA, USA). The protein concentration in the lysates was quantified using a Pierce BCA kit (Thermo Fisher Scientific). The proteins were separated by sodium dodecyl sulfate–polyacrylamide gel electrophoresis (SDS-PAGE) using 4-12% gradient gel (Thermo Fisher Scientific) and transferred onto polyvinylidene difluoride (PVDF) membranes. After the transfer of proteins, the membrane was blocked with LI-COR blocking buffer at room temperature (RT) for 1 hr. The membranes were incubated with primary antibody at 4°C overnight followed by incubation with secondary antibody at RT for 60 min. All antibodies used in the western blot are listed in **Supplementary Table 2**. The blots were visualized using a LI-COR Odyssey DLx imager (LI-COR Biosciences, Lincoln, NE, USA). The blotting images were processed and finalized by Image Studio Lite Ver 5.2. (LI-COR Biosciences).

Membranes were blocked with mouse anti-PPARα mAb (Thermo Fisher Scientific), mouse anti-CPT1A mouse mAb (Abcam), rabbit anti-CPT2 mAb (Abcam), and β-actin mAb (Sigma Cat. A3854) and then incubated with primary antibodies and IRDye^®^ Secondary Antibodies (Li-COR). The blots were visualized with chemiluminescence, and the densitometry of the blots was detected using a LI-COR Odyssey Fc imager (LI-COR Biosciences, Lincoln, NE, USA). The fluorescence intensity was evaluated using Image Studio Lite Ver 5.2.

### Indirect calorimetry, physical activity, and food intake

Indirect calorimetry was performed in an automated Phenomaster, TSE. Prior to the experiments, mice were acclimatized in the chambers for at least 24 hours. The mice were allowed free access to food and water during the measurement. Locomotor activity, oxygen consumption, and other parameters of indirect calorimetry, as well as food intake, were continuously monitored for 48 hours (16).

### Cell cultures

Peripheral blood mononuclear cells (PBMCs) were isolated using the Ficoll gradient method. The PBMCs were cultured in the RPMI complete medium (RPMI 1640 medium supplemented with 10% Fetal Bovine Serum, 1U of penicillin, 1 µg/ml of streptomycin, 0.5 mM of sodium pyruvate, and 10 mM of HEPES) containing 10 ng/mL of recombinant murine macrophage-colony stimulating factor (rmM-CSF) (PeproTech, Westlake Village, CA, USA) at 37°C for a 48 h (17), then the differentiated macrophages (monocyte-derived macrophages; MDMs) were harvested, and the quality was evaluated by flow cytometry. The samples with more than 90% CD11b+F4/80+ were used for subsequent experiments.

### Quantification and statistical analysis

Statistical analysis was performed by using GraphPad Prism 9.0 software (GraphPad Software, San Diego, CA, USA). The normal distribution of data was determined by the Shapiro-Wilk normality test. For statistical comparisons, student t-test or one-way ANOVA with Tukey’s analysis were used to compare normally distributed variables. Non-normal distributed data were compared by the Mann-Whitney U test (between two groups) or the Kruskal-Wallis test (between multiple groups). P < 0.05 was considered significant. Numbers per group in the figure legends refer to the number of mice per group.

## Results

### Myeloid lineage-specific ACE overexpression attenuates atherosclerosis accompanied by increasing Ly-6C^lo^ monocytes

**Figure 1:**
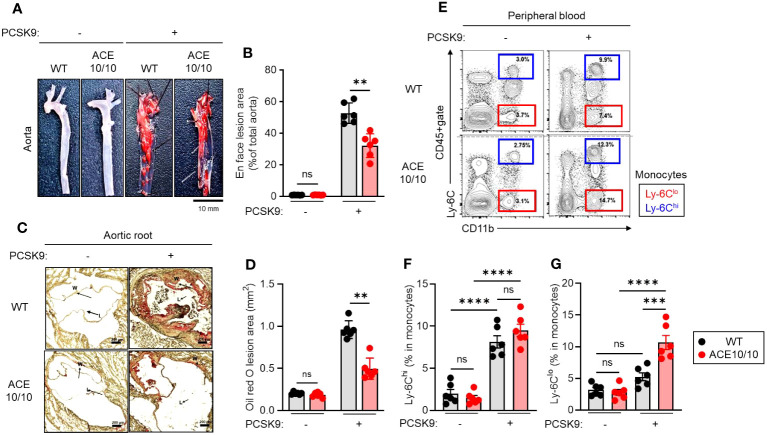
Myeloid lineage-specific ACE overexpression attenuates atherosclerosis accompanied by an increase in Ly-6C^lo^. Both WT and ACE10/10 mice with or without PCSK9-AAV-induced atherosclerosis. Pathological and immunological analyses were performed on each mouse. **(A, B)** Lipid deposition in the aorta. The deposited lipid plaques were stained with Sudan IV. **(A)** Representative pictures of lipid deposition in the aorta. Bar=10 mm. **(B)** Cumulative percentage values of lesion areas. **(C, D)** Histological analysis of aortic root with Oil Red O staining. **(C)** Representative picture of histology. Arrows indicate lipid deposition (W: wall; L: lumen, Bar: 200 µm). **(D)** Cumulative values of lesional areas (mm^2^). **(E–G)** Characterization of monocytes in peripheral blood (PB). PB was collected from each mouse and the monocyte population was analyzed by flow cytometry. **(E)** Representative plots of Ly-6C^hi^ and Ly-6C^lo^ monocyte populations. **(F, G)** Cumulative percentage values of Ly-6C^hi^ **(F)** and Ly-6C^lo^ **(G)** monocytes, respectively. The monocyte populations were identified by following Ly-6C expression in CD11b+cells in CD45+gate. The data were shown as representative or mean ± SEM of six samples in two independent experiments. Non-parametric t-test was used to analyze data for significant differences. Values of *p < 0.05, **p < 0.01, ***p < 0.001, and ****p<0.0001 were regarded as significant. ns, not significant.

First, we established an AAV-PCSK9-induced atherosclerosis model and performed pathological evaluation in both WT and ACE10/10 mice (**Figure 1A**). After 18 weeks of an atherogenic diet, AAV-PCSK9-inoculated mice generated atherosclerosis, and a large amount of plaque deposition was observed in the aortic wall compared with a control group. The grade of plaque deposition was evaluated by the occupancy of the lesional area in the aorta. ACE10/10 mice showed a significantly small lesional area (32.1 ± 2.51%) compared with that of WT mice (52.7 ± 2.16%) (**Figure 1B**). Histological analysis of aortic root showed that oil-red O-stained plaque burden was significantly larger in WT mice (0.961 ± 0.035 mm^2^) compared to ACE10/10 mice (0.497 ± 0.042 mm^2^) (**Figures 1C, D**). Interestingly, we found a significant increase in the Ly-6C^lo^ monocyte (CD45+CD11c+Ly-6C^lo^) population in ACE10/10 mice (10.6 ± 0.90%) compared to that of WT mice (5.25 ± -0.51%) with atherosclerosis. This population was greatly increased in ACE10/10 mice with atherosclerosis compared to the control condition, while WT mice had a similar size of population between control and atherosclerosis conditions (**Figures 1E, F**). On the other hand, Ly-6C^hi^ monocytes (CD45+CD11c+Ly-6C^hi^), which are classical inflammatory monocytes, were significantly increased in both WT and ACE10/10 in atherosclerosis compared to the control condition; however, the populational size was comparable between these two strains (**Figures 1E, G**).

Thus, myeloid lineage-specific ACE overexpression alters the populational balance of peripheral blood circulating monocytes in atherosclerosis.

### ACE overexpression enhances lipid metabolism in monocyte-derived macrophages

**Figure 2:**
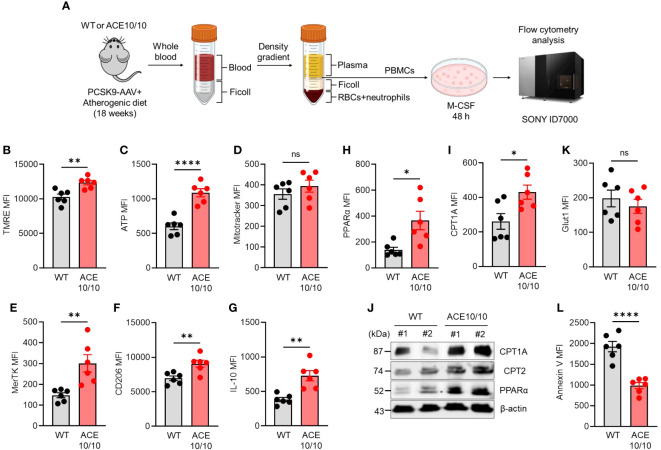
ACE overexpression triggers functional alteration in monocytes which are conserved in subsequently differentiated macrophages in atherosclerosis. **(A)** Experimental design of functional analysis of monocytes-derived macrophages (MDMs). The peripheral blood samples were collected from both WT and ACE10/10 with PCSK9-AAV-induced atherosclerosis. The peripheral blood mononuclear cells (PBMCs) were isolated by the density gradient method and the cells were cultured with M-CSF for 48 h to gain MDMs. The MDMs were used for analysis. **(B–I, K)** Functional characterization of MDMs. The expression levels of TMRE **(B)**, ATP **(C)**, Mitotracke **(D)**, MerTK **(E)**, CD206 **(F)**, IL-10 **(G)**, PPARα **(H)**, CPT1A **(I)**, and Glut 1 **(K)** were represented by following mean fluorescence intensities (MFIs). **(J)** WT image of PPARα, CPT1A, and CPT2 in MDMs. **(L)** The analysis of apoptotic status by Annexin V staining in flow cytometry. The MDM population was identified at CD11b+F4/80+ in each flow cytometry analysis. The data were shown as representative or mean ± SEM of six samples in two independent experiments. Non-parametric t-test was used to analyze data for significant differences. Values of *p < 0.05, **p < 0.01, ***p < 0.001, and ****p<0.0001 were regarded as significant. ns, not significant. Created with BioRender.com.

Next, we performed functional characterizations of monocytes in atherosclerosis in WT and ACE10/10 mice using an *in vitro* differentiation system. Monocytes were isolated from the peripheral blood of atherosclerosis mice, then the cells were differentiated into macrophages (MDMs) by treatment with M-CSF. The MDMs were subjected to functional characterizations (**Figure 2A**). We investigated mitochondrial membrane potential (ΔΨ) and intracellular ATP level by staining with tetramethylrhodamine ethyl ester (TMRE) and BioTracker ATP-Red, respectively. Both mitochondrial membrane potential and ATP levels were significantly increased in ACE10/10-originated MDMs compared to that of WT (**Figures 2B, C**). However, the numbers of mitochondria detected by Mitotracker were comparable between two different origins of samples (**Figure 2D**).

The polarization status was evaluated by following the expressions of specific parameters. The reparative macrophage (M2-like) markers, such as MER Proto-Oncogene, Tyrosine Kinase (MerTK), CD206 (also known as mannose receptor C type 1; MRC1), and interleukin (IL)-10 expressions were all significantly increased in ACE10/10-originated MDMs compared with those of WT MDMs (**Figures 2E–G**). Furthermore, PPARα and CPT1A expressions were both significantly increased in ACE-overexpressed MDMs compared to those of WT MDMs, implying that lipid metabolism was enhanced by ACE overexpression in MDMs (**Figures 2H, I**). These protein expressions were also confirmed by WB together with CPT2, which is another player in lipid metabolism, and all of the investigated three proteins in the lipid metabolic chain were elevated in their expression by ACE overexpression in MDMs (**Figure 2J**). On the other hand, glucose transporter 1 (glut 1) expressions were comparable between WT and ACE10/10-originated MDMs (**Figure 2K**). Unexpectedly, we found that the frequency of apoptotic cells was significantly decreased in ACE-overexpressed MDMs compared to WT MDMs, indicating decreased expression of cell surface apoptotic marker phosphatidylserine (PS) which binds with annexin V (**Figure 2L**).

Taken together, ACE overexpression modifies the character of monocytes which is subsequently reflected in the enhanced metabolic function and reparative-polarized status of differentiated macrophages in atherosclerosis.

### ACE overexpressing myeloid cells improves systemic energy expenditure

**Figure 3:**
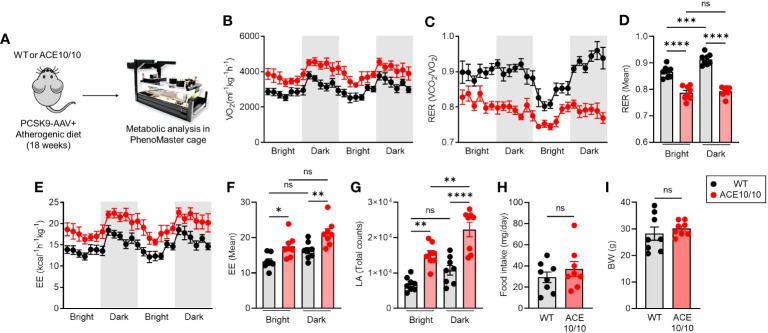
ACE overexpressed myeloid cells enhance systemic energy expenditure. **(A)** Experimental design of analysis for metabolic status in atherosclerosis mice. PCSK9-AAV-induced atherosclerosis was induced in both WT and ACE10/10 mice. The metabolic parameters were analyzed using phenomaster cages. All analyzed data were represented in **(B)** whole-body oxygen consumption rate (VO_2_), **(C, D)** respiratory exchange ratio (RER), **(E, F)** Energy expenditure, **(G)** locomotor activity, **(H)** daily food intake, and **(I)** body weight. The data were shown as mean ± SEM of eight samples in two independent experiments. Non-parametric t-test or one-way ANOVA was used to analyze data for significant differences. Values of *p < 0.05, **p < 0.01, ***p < 0.001, and ****p<0.0001 were regarded as significant. ns, not significant.

Although we revealed the protective phenotype of ACE10/10 mice in atherosclerosis based on enhanced lipid metabolism as well as the tissue-repairing function in macrophages, its influence on the systemic level is still unknown. Therefore, we decided to investigate the energy demand and activity based on the individual level. Atherosclerosis condition was established in both WT and ACE10/10 mice using the AAV-PCSK9 system, and then the metabolic parameters, food intake, and basal activities were analyzed in each mouse (**Figure 3A**). An analysis using indirect calorimetry (IC) revealed that the oxygen consumption rate (V_O2_) was significantly increased in ACE10/10 mice compared with WT mice in atherosclerosis condition (**Figure 3B**), while the respiratory exchange ratio (RER; V_CO2_/V_O2_) was significantly downregulated in ACE10/10 mice compared with WT mice (**Figure 3C**). The difference in RER was not influenced by circadian rhythms (**Figure 3D**). These results indicated that ACE10/10 mice preferred lipid oxidation rather than carbohydrate and protein utilization in energy production (**Figures 3C, D**) (18, 19). Moreover, energy expenditure (EE) and locomotor activity were both significantly higher in ACE10/10 mice compared to those of WT mice throughout the day (**Figures 3E–G**). Surprisingly, there was no difference in daily food intake and body weight (BW) between WT and ACE10/10 mice under atherosclerosis condition, which means that ACE10/10 mice effectively utilized fat contents by enhanced lipid metabolism even if it was limited in the monocytes/macrophages (**Figures 3H, I**).

## Discussion

In this study, we represented two crucial aspects of ACE overexpression in myelomonocytic lineage cells under atherosclerosis conditions. First, we revealed that ACE overexpression modified metabolism in peripheral blood monocytes, and this functional alteration was subsequently reflected in the differentiated macrophages (**Figure 2**). Second, we found that ACE overexpression in monocytes/macrophages influenced the systemic manner which was observed by increased energy demand in the ACE10/10 mice (**Figure 3**).

We have already reported that ACE-overexpressed macrophages provided a protective function in atherosclerosis conditions by enhanced lipid metabolism represented by promoted β-oxidation and PPARα upregulation in the lesional macrophages (7). However, there was an unrevealed part on how ACE overexpression influences myelomonocytic lineage cells during differentiation. Since peripheral blood circulating monocytes and splenic monocytes are precursors of atherosclerotic plaque-infiltrated macrophages (20), we investigated the influence of ACE overexpression in monocytes under atherosclerosis. Interestingly, *in vitro* MDM differentiation showed that ACE overexpression pre-established a functional modification in monocytes resulting in an increased lipid metabolism accompanied by polarization into reparative phenotype (**Figure 2**). This is a novel finding that ACE is a crucial factor in enhancing lipid metabolism not only for differentiated macrophages but also for peripheral blood circulating monocytes in atherosclerosis. This finding can be adapted to clinical diagnosis if ACE expression is enhanced in peripheral blood monocytes; the patients could expect a better outcome in atherosclerosis. In fact, we found that the frequency of Ly-6C^lo^ monocytes was increased in ACE10/10 mice with atherosclerosis (**Figures 1E, F**). We hypothesized that this population dominantly owed ACE-mediated metabolical and immunological functional changes greater than the Ly-6C^hi^ population when the cells were differentiated into macrophages in ACE10/10 mice. However, we have not yet performed a detailed analysis in a subset-dependent manner because the current MDM differentiation system used the whole monocyte population in the peripheral blood following Ly-6C expression. In future experiments, MDM differentiation using sorted Ly-6C^low^ and Ly-6C^hi^ monocytes, and comparison of MDM function in the two distinct resources may tell us the detailed differences in metabolic and immunological functions in atherosclerosis.

The importance of lipid metabolism of macrophages and macrophage-like cells has been reported for the progression and outcome of the other diseases. For instance, the immunopathogenesis of Alzheimer’s disease (AD) contains failing lipid handling and metabolism of brain microglia which possess similar differentiation origin and functional character with peripheral macrophage. In fact, we have already found that ACE10/10 mice showed highly resistant character in the AD model (9, 10). We revealed that ACE10/10 microglia captured and degraded amyloid beta (Aβ) as one of the potential benefits to attenuate AD symptom; however, there is a possibility that lipid metabolism is enhanced by ACE overexpression in the microglia.

It was another novel finding that systemic energy demand was increased in ACE10/10 mice in atherosclerosis (**Figure 3**). This is an unexpected finding because there was no report that the modification of lipid metabolism in myeloid cells alters systemic energy demand. On the other hand, we might be able to understand this change in ACE10/10 mice because macrophages are distributed to various tissues and organs, such as fat and liver which require high lipid metabolic reactions and energy consumption, rather than other immune cells (21, 22). To judge the crucial role of myelomonocytic lineage-specific ACE overexpression in systemic metabolic alteration, we believed that bone marrow transfer (BMT) was one of the indispensable experiential approaches.

As a molecular mechanism of enhanced lipid metabolism, we found that ACE overexpression triggered activation of the lipid metabolism chain by the upregulation of PPARα-CPT1A/2 in macrophages in this study and in a former report (**Figure 2**) (7). Since PPARα is one of the important transcription factors, PPARα agonist enhanced lipid metabolism in oleic acid (OA)-treated macrophages. In addition, ACE inhibitor abolished the upregulation of PPARα and CPT1A and attenuated the lipid metabolism in the ACE-overexpressed macrophages. These responses consequently increased ATP production by activating mitochondrial β-oxidation. Moreover, PPARα inhibition fails to induce macrophages to gain an immunological reparative character. These results suggested that PPARα and related lipid metabolic chains are important factors to regulate macrophages’ function and enhance ACE expression, which might have a crucial role in the PPARα-mediated metabolic alteration in the macrophages. However, we have not yet revealed the detailed mechanism of how ACE regulates PPARα expression in macrophages.

Although there are still some unrevealed parts, our result highlights the importance of ACE expression in the lipid metabolism of macrophages. These findings also provide a possible focus that regulating ACE may develop future therapeutic approaches for various diseases with lipid metabolism defect and inflammation.

## Data availability statement

The original contributions presented in the study are included in the article/**Supplementary Material**. Further inquiries can be directed to the corresponding authors.

## Ethics statement

The animal study was approved by Institutional Animal Care and Usage Committee (IACUC) of Cedars-Sinai Medical Center. The study was conducted in accordance with the local legislation and institutional requirements.

## Author contributions

DC: Conceptualization, Data curation, Formal Analysis, Funding acquisition, Investigation, Methodology, Project administration, Supervision, Validation, Writing – original draft, Writing – review & editing. SS: Conceptualization, Data curation, Formal Analysis, Investigation, Methodology, Validation, Writing – original draft, Writing – review & editing. LX: Conceptualization, Methodology, Software, Writing – review & editing. WF: Conceptualization, Data curation, Investigation, Methodology, Resources, Writing – review & editing. XL: Investigation, Methodology, Resources, Validation, Writing – review & editing. FA: Investigation, Methodology, Resources, Writing – review & editing. PJ: Conceptualization, Data curation, Investigation, Methodology, Software, Writing – review & editing. TS: Investigation, Methodology, Writing – review & editing. MC: Investigation, Methodology, Resources, Writing – review & editing. EB: Investigation, Methodology, Resources, Writing – review & editing. JG: Investigation, Methodology, Resources, Writing – review & editing. AD: Conceptualization, Methodology, Resources, Software, Writing – review & editing. DO-D: Funding acquisition, Investigation, Methodology, Resources, Writing – review & editing. ZK: Conceptualization, Investigation, Methodology, Resources, Writing – review & editing. CR: Investigation, Methodology, Resources, Supervision, Writing – review & editing. FC: Conceptualization, Methodology, Resources, Software, Writing – review & editing. KB: Conceptualization, Funding acquisition, Project administration, Resources, Supervision, Validation, Writing – review & editing.
