# Supplementary material for: Myeloid cell ACE shapes cellular metabolism and function in PCSK-9 induced atherosclerosis
Source: Front Immunol. 2023 Oct 20;14:1278383. doi: 10.3389/fimmu.2023.1278383 (PMC10623052; doi:10.3389/fimmu.2023.1278383)
Supplement: Supplementary file 1 [file Table_1.docx]

Supplementary Material

# Supplemental Table 1. Antibody list in flow cytometry analysis

**Supplemental Table 2. Antibody list in western blot**
